# Medical Administration in the Trenches

**Published:** 1915-06-19

**Authors:** 


### CONSERVANCY METHODS ON ACTIVE SERVICE.

### Medical Administration in the Trenches.

THE problems involved in maintaining the fighting efficiency of large bodies of troops are, it is safe to say, practically unknown to the ordinary general practitioner, and even the possessor of a diploma in public health may be stricken with a feeling of helplessness when faced with the difficulties of camp sanitation. The duties of army doctors in relation to the care of the sick and wounded are naturally those which fire most readily the imagination of the layman, and conceptions of field ambulances, hospital trains and ships are easily formed; but medical organisation and administration in the field claim a far wider interpretation.

The precise *rôle* of the medical service in relation to war has been open to misconception, and it is useful to recall Sir Evelyn Wood's definition of the essential duty of the medical officer. He enunciated this as follows:—"Medical officers are trusted staff officers whose duty it is to guard the troops against disease, with the object of increasing fire effect by raising the fighting value of the troops and reducing hospital transport and establishments." Thus the primary object of the army medical service is the same as that of the army services—namely, the defeat of the enemy with the smallest loss and in the shortest time. Individual considerations of suffering or death cannot be permitted to retard the prosecution of this object. In military history we read of instances of forced marches being carried out with apparently unspeakable callousness and numbers of dying men being left by the wayside. But the purchase of immediate victory at small cost has to compensate for this relentless severity. This is exemplified in one case described by Fortescue during Marlborough's last campaign, when "hundreds dropped, and many died there and then; but they were left where they lay." The accepted practice in medicine and surgery is to remove the cause of pain rather than the pain itself; and as the cause of suffering in war is the state of war itself, victory is the one remedy for the suffering and for the disease. Anything, therefore, which can make for victory is to be permitted to override medical considerations.

The relation of the medical to the combatant branch is a matter which needs to be clearly borne in mind by medical officers. At the present time, when there are so many medical officers working for the Army who are but imperfectly acquainted with the limitations of their department and the wide powers and responsibility of commanding officers, there is apt to occur a feeling of resentment on the part of a medical officer when his advice on some seemingly essential point is rejected by the combatant superior officer. Hard cases, no doubt, crop up now and again, and it is possible even for commanding officers to err; but it is only insubordination for the medical officer to sulk or to abstain from tendering further advice which it is his duty to give.

Such considerations as these receive the adequate attention which is their due in a manual for R.A.M.C. officers that has just been published.* The author is Major Lelean, who is Assistant Professor of Hygiene at the Royal Army Medical College. The book, though small, is an important one, and a more practical volume, considering its size, could hardly be produced. In substance it consists of lectures delivered to newly-joined officers for the purpose of giving them an insight into the mysteries of sanitation on active service. It may easily be imagined, as the author states in his modest prefatory remarks, that the task of preparing amid the exigencies of war-time even such an unpretentious volume as this has been beset with difficulties. Though Major Lelean seeks to forestall an anticipated "avalanche of adverse criticism," it will be found, we doubt not, that his sins of omission and commission are few, and are compensated by the very concise and practical nature of his lectures. To the layman even a perusal of these pages is an education which he can apply with benefit to his civil mode of life. The advantages and the physical properties of various clothing materials and the "points" of a good boot are obvious instances.

The term "sanitation," it need hardly be mentioned, covers a very great deal more than conservancy methods, but, in view of the unparalleled circumstances of the present war, the subject becomes of interest even to the most unimaginative householder, who must dimly appreciate the immensity of the task of dealing with the waste products of enormous numbers of men and animals under unfavourable conditions. The perfection of our municipal sanitary organisations tends to engender a considerable degree of igorance and helplessness in the individual. A quotation from the chapter concerned sums up this attitude: "The acquaintance of an ordinary householder with conservancy methods consists in the daily pulling of a handle and a weekly listening to the opinion of the dustman on the dryness of his job."

The author allows himself a short humorous digression in tracing the evolution of burial methods of disposal from the surface methods of our Simian ancestors to the demands of "hyperosmic landlords," and then proceeds to give a description of the regulation trenches and the usual precautions against flies, etc., to say nothing of "camp butterflies," which is the euphemism attached to bits of paper blown out of the trenches. A thorough consideration is given to the alternative method of disposal—namely, incineration—which on active

* *Sanitation in War.* By Major P. S. Lelean, F.R.C.S., D.P.H., R.A.M.C. With an Introduction by Surgeon-General Sir Alfred Keogh, K.C.B., F.R.C.P. Illustrated. London: J. and A. Churchill. 1915. Price 5s.

service obviates so many difficulties and dangers that the economic objection that the burning of organic matter involves a drain of nitrogen from the soil must be overruled. In peace-time this view is one which has considerable practical importance, and is met to some extent in sewage farms and in certain Colonial traditions regarding the obligation of a guest to pay a practical visit to his host's vegetable garden.

Incinerators of various types and their working are described, and here it is interesting to note that both camp and municipal incinerators have been objected to by members of the public much more often on account of a supposed offence to the æsthetic senses than by reason of actual malodorousness. The suspicion that filth is being cremated is sufficient to turn the smell of burning wood into the "intolerable effluvium of burning matter of an indescribably objectionable nature," as in an instance quoted by the author; whereas it is a matter of fact that the odour of burning organic matter is much the same, whatever its nature, so long as it is fresh and undried. To the subject of the disposal of corpses by incineration only brief reference is made. The method is apparently satisfactory and practicable, and is said to have been employed by some of the armies now in the field. Our own dead, it may be supposed, have not been submitted to this process, for the writer suggests that public sentiment would not permit of its adoption. The more this point is thoughtfully considered the more illogical does it appear under the circumstances of the present war.

### Continental Conservancy.

The inferiority of Continental practice in conservancy matters, compared with those in vogue in any well-managed British town, makes the disadvantages of campaigning in a friendly country somewhat acutely felt, and sanitary officers need to display a great deal of tact. A brief description of the municipal sanitation of a typical town now occupied by our troops is, therefore, appropriately given in this book. A superficial observer may readily receive the impression, after inspection of such methods as are commonly to be found on that part of the Continent in question, that our own conservancy methods are unnecessarily refined and have led to much waste of money. But reference to the statistics of enteric fever in the capitals of the chief belligerent countries, as set out in a useful diagram on page 215, will soon set at ease the mind of the sceptical ratepayer. The amount of enteric fever in Paris is approximately three times that in London; in Brussels more than twice as much; in Petrograd the figure is ten times as great; while in Berlin even it is higher by a third. The disparity shown by the French figures would certainly be more marked there than with us if compulsory notification were in force. Further, as Major Lelean points out, there is a very large amount of sickness among French children, and the liability to infection of water supplies by leakage into them of cesspit contents justifies the view that many of these cases of sickness may be due to enteric, which in children is often mild and difficult to diagnose. Hence it may be supposed that a considerable proportion of the adult population is immune to enteric itself, but there must also be a large number of carriers going about. And this factor makes the area the more dangerous to our own soldiers. The conclusion to be drawn, then, is that the expenditure on our conservancy methods is fully vindicated.

### The Water Problem for the Allies.

In passing a reference may be permitted to a method of conservancy used in the trenches—but only for a very short time!—of which tales are told in clubs and smoke-rooms. This was a reversion to the Chinese stink-pot, and consisted in lobbing bully-beef tins full of excreta into neighbouring enemy trenches. Naturally reprisals ensued at once, and were found so unpleasant that by mutual consent the method was abandoned. The consideration of water supplies and methods of purification occupies necessarily an important position in Major Lelean's manual. The difficulties which have been already overcome were sufficiently great to tax fully the ingenuity of our experts, but it appears certain that when our long-hoped-for advance begins our Forces will have to face a water problem of the utmost gravity. The reasons for this apprehension are given in a concluding section of the chapter, and a useful map illustrates the geological formations in relation to the water supplies of North-East France and of Belgium. It is gratifying to note that among the numerous methods of sterilising water which have been used on active service, that in which bleaching-powder is used as the source of chlorine has been satisfactorily developed. For imperative reasons reticence is maintained regarding the full details which have been worked out in the present war.

A bare reference must suffice for the other highly important lectures in this admirable manual. The *rôle* of the fly in the dissemination of disease is now rapidly becoming common knowledge. It is difficult to realise, though the author gives the quotation, that the fly has been described as a "dipterous angel dancing light attendance upon Hygeia," for now everyone agrees in denouncing this insect as "a winged sponge flying hither and thither to fulfil the behests of Contagion." The practical chapter on insects in war is decidedly one of the most useful, and should appeal strongly to those at home.

The titles of some of the other lectures—Anti-typhoid Inoculation, The March, Sickness in the Army—will indicate that in this manual the medical officer will have not only a helpful text-book while training, but also a portable guide.

It is interesting to learn that the most common apprehension among men going into action is that of death from hæmorrhage, which they firmly believe can be always arrested by the medical officer if he be at hand. Hence the sense of security afforded to the men by the proximity of the medical officer is a valuable psychological factor, which may possibly be allowed to outweigh the risk of the serious disaster which the loss of its doctor at a critical juncture always is to a Red Cross unit.

